# Using Ribonucleoprotein-based CRISPR/Cas9 to Edit Single Nucleotide on Human Induced Pluripotent Stem Cells to Model Type 3 Long QT Syndrome (*SCN5A*^±^)

**DOI:** 10.1007/s12015-023-10602-5

**Published:** 2023-08-31

**Authors:** Ning Ge, Min Liu, Rui Li, Nicholas M. Allen, Joseph Galvin, Sanbing Shen, Timothy O’Brien, Terence W. Prendiville

**Affiliations:** 1 Regenerative Medicine Institute, School of Medicine, College of Medicine, Nursing and Health Science, University of Galway, Galway, Ireland; 2 Department of Pharmacology, Northwestern University Feinberg School of Medicine, Chicago, IL USA; 3 Department of Physiology, College of Life Science, Hebei Normal University, Shijiazhuang, China; 4 Lambe Institute for Translational Research, University of Galway, Galway, Ireland; 5 Department of Paediatrics, University of Galway, Galway, Ireland; 6 Mater Misericordiae University Hospital, Eccles St., Dublin 7, Ireland; 7 FutureNeuro, The SFI Research Centre for Chronic and Rare Neurological Diseases, Royal College of Surgeons in Ireland, Dublin, D02 Ireland; 8 National Children’s Research Centre, Children’s Health Ireland at Crumlin, Dublin 12, Ireland

**Keywords:** Long QT syndrome, Induced pluripotent stem cells, CRISPR/Cas9, Electroporation, Disease modelling

## Abstract

Human induced pluripotent stem cells (hiPSCs) have been widely used in cardiac disease modelling, drug discovery, and regenerative medicine as they can be differentiated into patient-specific cardiomyocytes. Long QT syndrome type 3 (LQT3) is one of the more malignant congenital long QT syndrome (LQTS) variants with an *SCN5A* gain-of-function effect on the gated sodium channel. Moreover, the predominant pathogenic variants in LQTS genes are single nucleotide substitutions (missense) and small insertion/deletions (INDEL). CRISPR/Cas9 genome editing has been utilised to create isogenic hiPSCs to control for an identical genetic background and to isolate the pathogenicity of a single nucleotide change. In this study, we described an optimized and rapid protocol to introduce a heterozygous LQT3-specific variant into healthy control hiPSCs using ribonucleoprotein (RNP) and single-stranded oligonucleotide (ssODN). Based on this protocol, we successfully screened hiPSCs carrying a heterozygous LQT3 pathogenic variant (*SCN5A*^±^) with high efficiency (6 out of 69) and confirmed no off-target effect, normal karyotype, high alkaline phosphatase activity, unaffected pluripotency, and *in vitro* embryonic body formation capacity within 2 weeks. In addition, we also provide protocols to robustly differentiate hiPSCs into cardiomyocytes and evaluate the electrophysiological characteristics using Multi-electrode Array. This protocol is also applicable to introduce and/or correct other disease-specific variants into hiPSCs for future pharmacological screening and gene therapeutic development.

**Graphical Abstract:**

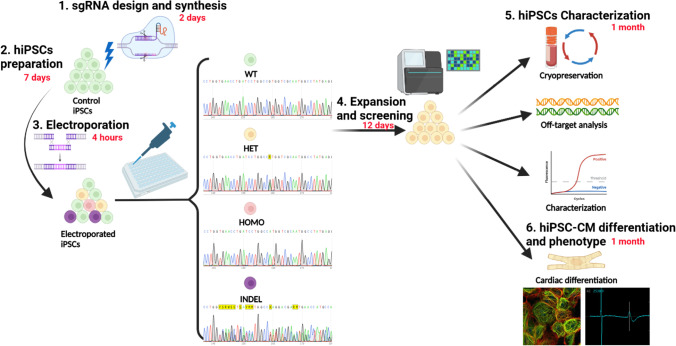

**Supplementary Information:**

The online version contains supplementary material available at 10.1007/s12015-023-10602-5.

## Introduction

**Fig. 1:**
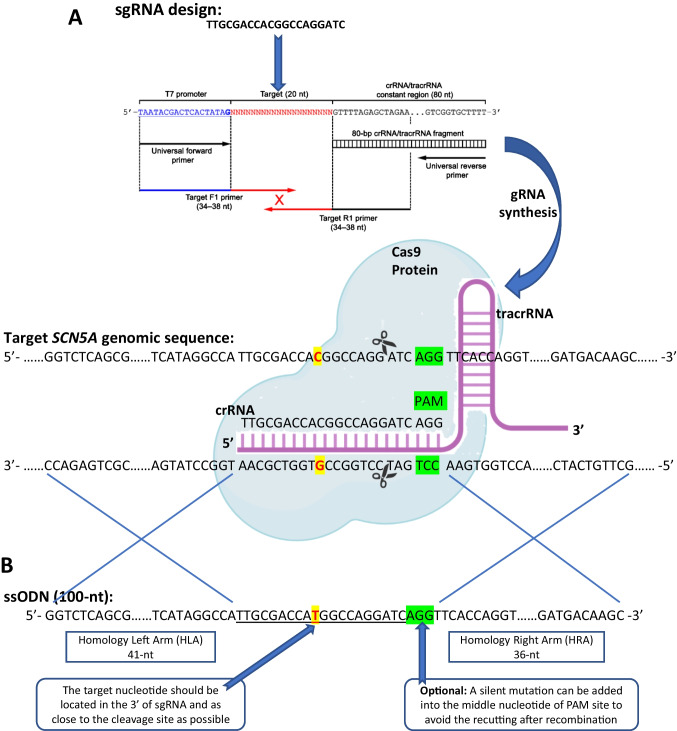
sgRNA and ssODN design for single nucleotide editing. (A) sgRNA design and in vitro synthesis. Firstly, the target nucleotide (pathogenic variant of interest) is located in the 3’ of sgRNA and close to the Cas9 cleavage site which is normally at 3-4nt upstream (5’ side) of the protospacer adjacent motif (PAM). Then a commercial kit was used to synthesize the CRISPR system consisting of a target complementary CRISPR RNA (crRNA) and an auxiliary trans-activating crRNA (tracrRNA). (B) ssODN design for homology direct repair (HDR) after Cas9-incued DNA double-stranded breaks (DSB). The 100-nt ssODN template contains a 41-nt homology left arm (HLA) and a 36-nt homology right arm (HRA). It is also recommended to introduce a synonymous (silent) mutation at PAM to avoid re-cutting after recombination

**Fig. 2:**
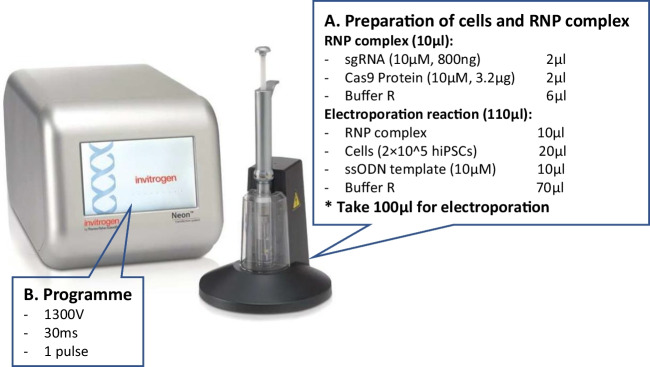
Electroporation of RNP complex into hiPSCs using Neon Transfection System. (**A**) Prepare the electroporation reaction mix of RNP (consisting of sgRNA and Cas9 protein), cells and ssODN. Transfer 100 μl of the mixture using the Neon Pipette and connected inside of the Neon Pipette Station. (**B**) Select Programme ‘1300 V, 30 ms, 1pulse’ and press ‘Start’ to perform the electroporation

**Fig. 3:**
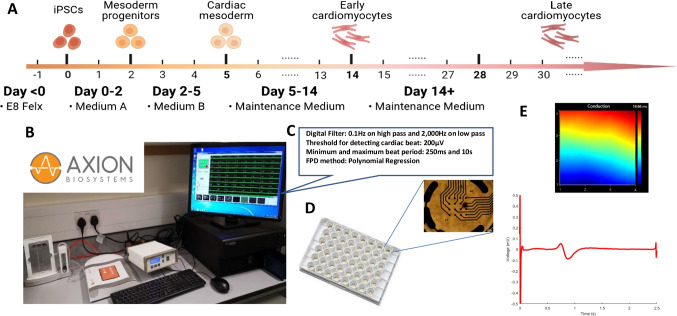
HiPSC-CM differentiation and MEA recording. (**A**) Schematic diagram of the cardiac differentiation protocol from hiPSCs. (**B**) The MEA platform (Axion BioSystems) used in our research. (**C**) The parameter setting in the AxIS software in MEA data recording. (**D**) HiPSC-derived cardiomyocytes plated on CytoView MEA plates. (**E**) The typical conduction velocity and the field potential on the MEA

Congenital long QT syndrome (LQTS) is the most common primary inherited arrhythmia syndrome that can contribute to risk of sudden death and occurs in an estimated 1 per 2,000 of the general population. Fifteen autosomal dominant genes have been identified to be associated with different LQTS sub-types [1] and single nucleotide substitutions (missense). Small insertion/deletions (INDEL’s) are the predominant mode of genetic variants identified in LQTS genes. LQTS type 3 (LQT3) is the most malignant sub-type of congenital LQTS with a gain-of-function (GOF) variant of the I_Na_ channel (encoded by *SCN5A*) resulting in impaired Na_V_1.5 inactivation [2]. This results in a peaked T wave morphology on the patient’s electrocardiogram after a prolonged isoelectric ST segment [3, 4] (Figs. 1, 2, and 3).

The human induced pluripotent stem cells (hiPSCs) have a major advantage for personalized medicine because they can be re-programmed from the human somatic cells of a particular patient without the ethical and technical challenges of utilising embryonic stem cells (ESCs). In addition, the hiPSCs are able to expand indefinitely *in vitro* due to their self-renewal capacity, and then differentiate into a variety of cell types, including cardiomyocytes. HiPSCs have tremendous potential to generate research models that faithfully reflect patient pathophysiology and underlying cellular pathological mechanisms [5, 6]. Despite these advantages, a major challenge in the field of disease modelling using hiPSCs is discriminating between abnormal *in vitro* disease phenotype, and background heterogeneity [7]. To overcome this hurdle, appropriate isogenic hiPSC lines are considered the most accurate platform for disease modelling studies and controlling for genetic background effect [8, 9]. With this goal in mind, isogenic hiPSC lines can be generated using genome engineering methods.

Currently, there are three major genome editing techniques: zinc finger nucleases (ZFNs), transcription activator-like effector nucleases (TALENs) and Clustered Regularly Interspaced Short Palindromic Repeats (CRIPSR). Both ZFNs and TALENs use mega-nuclease (*Fok*I endonucleases) proteins to recognize and bind to specific DNA sequences through a protein-DNA interaction [10–12]. However, their accuracy and specificity are less than CRISPR/Cas9, additionally, the protein design, synthesis, and validation are more time consuming, technically challenging, inefficient, and relatively cytotoxic which may limit their routine use [13–16]. Consequently, the CRISPR/Cas9 system using a guide RNA (sgRNA) and CRISPR-associated (Cas) nuclease has become the dominant technique for genome editing due to its high efficiency and accuracy, cost-effectiveness and easy to use [17–20].

In the CRISPR/Cas9 system, a conserved 3 bp DNA sequence (NGG) at the 3’ end of sgRNA called protospacer adjacent motif (PAM) reading in the opposite direction to the RNA–DNA hybrid is essential for identifying the target DNA sequence by Cas9. Once specific site recognition by sgRNA occurs, Cas9-induced DNA double-stranded breaks (DSB) occurs. Following the DSB in the host cell, the damage is joined by one of two major endogenous cellular repair pathways, Non-Homologous End Joining (NHEJ) and Homology Directed Repair (HDR) [21]. NHEJ is the dominant pathway with higher efficiency and can be widely used for research purposes in the development of knock-out strategies to study loss-of-function (LOF) of an encoded protein [22]. Despite the NHEJ pathway being the dominant repair mechanism *in vivo*, it is prone to introducing error in DNA repair. HDR-mediated genome editing shows extremely high accuracy and error-free repair through the presence of either a homologous chromosomal DNA or an exogenous DNA template. Therefore, HDR is the preferred and most precise method in introducing/correcting specific pathogenic variants *in vitro* to recapitulate/rescue the disease genotype and phenotype, but has a relatively low efficiency that remains a research challenge [23].

The most efficient and widely used non-viral delivery process in CRISPR/Cas9 system is electroporation, which can deliver RNA/DNA/proteins into the target cells rapidly [24]. In addition, Cas9 protein and sgRNA can form a DNA-free format—Ribonucleoprotein (RNP) complex [25, 26], which has a lower molecular weight than plasmid DNA and can evade the inhibition by cellular mRNA mechanisms [27–30]. This allows the genome editing components to be transiently expressed and then quickly cleared from the cells by degradation, thus facilitating higher editing efficiency and fewer potential off-target effects in a variety of cell types [31]. To achieve optimal gene therapy technology, we also need to develop more effective delivery tools as well as more effective sgRNAs [32]. In addition to Cas9/gRNA, single-stranded oligodeoxynucleotides (ssODNs) must be available at the DNA repair site as a template for HDR pathway [33].

In this protocol, we used the publicly available design tool (https://zlab.bio/guide-design-resources) and selected the best candidate sgRNA that had a high on-target potential with low off-target risk for genome editing, and designed the ssODN for our specific target variant of interest. We then electroporated the RNP complex (sgRNA/Cas9) and ssODN together into hiPSCs by using Neon Transfection. We also differentiate these hiPSCs into cardiomyocytes using a very simple protocol. These protocols can also be applied for correcting disease-associated pathogenic variants for gene therapy.

## Materials

### sgRNA Synthesis and Analysis

- PCR oligonucleotides for sgRNA synthesis and ssODN were ordered from Integrated DNA Technologies
- PCR primers for off-target analysis were ordered from Eurofins Genomics
- GeneArt™ Precision gRNA Synthesis Kit (Invitrogen, #A29377)
- TrueCut Cas9 Protein v2 (Invitrogen, #A36496)
- TE Buffer (Invitrogen, #12090015)
- UltraPure DNase/RNase-free Water (Invitrogen, #10977–035)
- Tris Base (Fisher, #BP152-1)
- Ethylenediaminetetraacetic acid disodium salt dihydrate (EDTA) (Sigma-Aldrich, #E5134)
- Acetic Acid (Sigma-Aldrich, #A6283)
- Sodium hydroxide (NaOH) (Sigma-Aldrich, #S5881)
- Agarose (Sigma-Aldrich, #A9539)
- SYBR Safe DNA Gel Stain (Invitrogen, #S33102)
- 100 bp DNA Ladder (New England Biolabs, #N3231)
- DNA Gel Loading Dye (6 ×) (New England Biolabs, #B7025)
- Low Range ssRNA Ladder (New England Biolabs, #N0364S)
- RNA Loading Dye (2 ×) (New England Biolabs, #B0363S)
- NEBuffer r3.1 (New England Biolabs, #B6003S)
- DNeasy Blood & Tissue Kit (Qiagen, #69504)
- Proteinase K (> 600mAU/ml; Qiagen, #19131)
- RNase A (100 mg/ml; Qiagen, #19101)
- 3 M NaOAc (Sigma-Aldrich, #567244)
- Isopropanol (Sigma-Aldrich, #I9516)
- Ethanol, Molecular Biology Grade (Fisher Scientific, #16606002)
- AllTaq Master Mix Kit (Qiagen, #203144)
- QIAquick PCR Purification Kit (Qiagen, #28104)

### *iPSC Culture and Cardiomyocyte Differentiation Medium*

- Essential 8™ Flex Medium (Gibco, #A2858501)
- Geltrex™ LDEV-Free, hESC-Qualified, Reduced Growth Factor Basement Membrane Matrix (Gibco, #A1413302)
- Matrigel Growth Factor Reduced Basement Membrane Matrix (Corning, #356230)
- KnockOut™ DMEM (Gibco, #10829–018)
- KnockOut™ Serum Replacement (Gibco, #10828028)
- TrypLE™ Select Enzyme (1 ×), no phenol red (Gibco, #12563–011)
- Gentle Cell Dissociation Reagent (STEMCELL Technologies, #100–0485)
- ROCK inhibitor / Y-27632 (STEMCELL Technologies, #72304)
- RevitaCell™ Supplement (Gibco, #A26445-01)
- DMEM/F-12, GlutaMAX Supplement (Gibco, #31331–028)
- L-glutamin (Gibco, #A2916801)
- MEM Non-Essential Amino Acid Solution (NEAA) (Gibco, #11140–035)
- Penicillin–Streptomycin (Gibco, #15070063)
- β-mercaptoethanol (Gibco, #31350010)
- PSC Cardiomyocyte Differentiation Kit (Gibco, #A29212-01), including Cardiomyocyte Differentiation Medium A (A29209-01), Cardiomyocyte Differentiation Medium B (A29210-01) and Cardiomyocyte Maintenance Medium (A29208-01)
- STEMdiff™ Cardiomyocyte Dissociation Medium (STEMCELL Technologies, #05026)
- STEMdiff™ Cardiomyocyte Support Medium (STEMCELL Technologies, #05027)
- Fibronectin bovine plasma (Sigma-Aldrich, #F1141)
- Dulbecco′s Phosphate Buffered Saline, with MgCl_2_ and CaCl_2_ (Sigma-Aldrich, #D8662)
- Dulbecco′s Phosphate Buffered Saline, no Calcium, no Magnesium (Gibco, #14190–144)

### iPSC and Cardiomyocyte Characterization Reagents

- Alkaline Phosphatase Staining Kit II (Stemgent, #000055)
- Tween-20 (Sigma-Aldrich, #655204)
- AggreWell 400 Plate (STEMCELL Technologies, #34815)
- RNeasy Mini Kit (Qiagen, #74104)
- QuantiNova Reverse Transcription Kit (Qiagen, #205411)
- Fast SYBR Green Master Mix (Applied Biosystems, #4385612)
- PCR Mycoplasma Test Kit I/C (PromoCell, #PK-CA91-1096)
- qRT-PCR primers for pluripotent markers (*OCT4*, *SOX2* and *NANOG*) and cardiac markers (including *ACTN2*, *TNNT2*, *MYL2*, *MYH6*, *MYH7*) were ordered from Eurofins Genomics

### Antibodies

- Rabbit anti-OCT-4A (IgG) primary antibody (Cell Signaling Technology, #2840)
- Rabbit anti-SOX2 primary antibody (Cell Signaling Technology, #3579)
- Rabbit anti-NANOG primary antibody (Cell Signaling Technology, #3580)
- Mouse anti-SSEA4 (IgG3) primary antibody (Cell Signaling Technology, #4755)
- Mouse anti-TRA-1–60 (IgM) primary antibody (Cell Signaling Technology, #4746)
- Mouse anti-TRA-1–81 (IgM) primary antibody (Cell Signaling Technology, #4745)
- Goat anti-SOX17 primary antibody (R&D Systems, #AF1924)
- Mouse anti-α-Actin, Smooth Muscle (α-SMA) primary antibody (Cell Marque Corp, #202 M-95)
- Rabbit anti-β3-Tubulin (TUJ1) (IgG) primary antibody (Cell Signaling Technology, #5568)
- Mouse anti-Troponin T primary antibody (ThermoFisher, #MS-295-P1)
- Mouse anti-α-Actinin primary antibody (Sigma-Aldrich, #A7811)
- Rabbit anti-MYL2 primary antibody (Proteintech, #10906–1-AP)
- Goat anti-mouse IgG (H + L) Alexa Fluor 555 secondary antibody (Cell Signaling Technology, #4409)
- Goat anti-rabbit IgG (H + L) Alexa Fluor 488 secondary antibody (Cell Signaling Technology, #4412)
- Donkey anti-goat IgG Fluor 488 secondary antibody (ThermoFisher, #A11055)

### Equipment

- Neon™ Transfection System (Invitrogen, #MPK5000)
- Neon™ Transfection System 100 µL Kit (Invitrogen, #MPK10096)
- Tissue culture plate, 6-well (Sarstedt, #83.3920.300)
- Tissue culture plate, 12-well (Sarstedt, #83.3921.300)
- Tissue culture plate, 24-well (Sarstedt, #83.3922.300)
- Tissue culture plate, 96-well (Sarstedt, #83.3924.300)
- 15 ml centrifuge tube (Sarstedt, #62.554.502)
- 50 ml centrifuge tube (Sarstedt, #62.547.254)
- Microcentrifuge tube, 1.5 ml (Sarstedt, #72.690.001)
- Cell Scraper (Sarstedt, #83.1831)
- Cryogenic tube (Thermo Fisher, #363401)
- Mr. Frosty Freezing Container (Thermo Scientific, #5100–0001)
- µ-slide 8 Well (ibidi Gmbh, #80826)
- NanoDrop™ 2000/2000c Spectrophotometers (Thermo Scientific, #ND-2000)
- Veriti Gradient Thermal Cycler (Applied Biosystems, #4375786)
- StepOnePlus™ Real-Time PCR System (Applied Biosystems, #4376600)
- Confocal Microscope FV1000 (Olympus)
- Maestro Original (Axion BioSystems)
- CytoView MEA 48 Plate (Axion BioSystems)

### Software and Online Tools

- UCSC Genome Browser, UCSC Genome Browser on Human: https://genome.ucsc.edu/cgi-bin/hgGateway?hgsid=582958475_8MBq3n1SNhypj2lRHFl3oCfRsrrd&redirect=manual&source=genome.ucsc.edu
- BLAST, human genome online tool: https://blast.ncbi.nlm.nih.gov/Blast.cgi
- Primer designing tool: https://www.ncbi.nlm.nih.gov/tools/primer-blast/ [34]
- CRISPR/Cas9 guide RNA (sgRNA) design tool on Integrated DNA Technologies: https://eu.idtdna.com/site/order/designtool/index/CRISPR_CUSTOM [18]
- SnapGene Viewer: https://www.snapgene.com/snapgene-viewer
- AxIS Navigator, CiPA Analysis Tool: https://www.axionbiosystems.com/products/mea/mea-software

## Methods

1. **sgRNA design •** **TIMING 1 day**
  1.1 Based on the patient’s pathogenic variant information (*SCN5A* c.1231G>A, exon 9), full genomic DNA sequencing can be found on UCSC Genome Browser.

- ‘Species’: Homo Sapiens
- ‘Human Assembly’: Dec.2013(GRCh38/hg38)
- ‘Position/Search Term’: *SCN5A* (ENST00000333535.9) – chr3:38548062-38649687 – Homo sapiens sodium voltage-gated channel alpha subunit 5
- ‘Sequence and Links to Tools and Databases’: Genomic Sequence (chr3:38,548,062-38,649,687).
- ‘Sequence Retrieval Region Options’: Select 5’ UTR Exons, CDS Exons, 3’ UTR Exons and Introns.
- ‘Sequence Formatting Options’: Exons in Upper case, everything else in Lower case.
- Click ‘Submit’ to download the gene sequence of *SCN5A.*
  1.2 Single-stranded guide RNA (sgRNA) was designed for introducing the *SCN5A* c.1231G>A variant using a publicly available design tool (https://zlab.bio/guide-design-resources) from Integrated DNA Technologies (IDT, USA).
- Click ‘IDT’
- ‘Design custom gRNA’ – ‘Species’: Homo sapiens, ‘Input format’: FASTA Sequence
- Pick a 201bp length of wild-type (WT) genomic DNA sequence (Human Gene *SCN5A* ENST00000333535.8) surrounding the pathogenic variant of interest:

>*SCN5A* gctcccccagACCCTCAGGTCCGCAGGGAAGATCTACATGATCTTCTTCATGCTTGTCATCTTCCTGGGGTCCTTCTACCTGGTGAACCTGATCCTGGCC  TGGTCGCAATGGCCTATGAGGAGCAAACCAAGCCACCATCGCTGAGACCGAGGAGAAGGAAAAGCGCTTCCAGGAGGCCATGGAAATGCTCAAGAAAGAA (*)

*  colour indicates the variant of interest.

- Click ‘DESIGN’
- Based on the on-target score, off-target score, and the flanking site, the best sgRNA was selected.

5’-TTGCGACCACGGCCAGGATC-3’ (-) PAM: AGG, On-target score: 57, Off-target score: 85.

1.3 A single-stranded donor oligonucleotide (ssODN) was designed based on the sgRNA to introduce the variant of interest via homology direct repair (HDR):

GGTCTCAGCGATGGTGGCTTGGTTTTGCTCCTCATAGGCCATTGCGACCA  GGCCAGGATC  TTCACCAGGTAGAAGGACCCCAGGAAGATGACAAGC (100-nt) (*)

*  colour indicates the variant of interest.  colour shows the PAM sequence of the chosen sgRNA.

1.4 Based on the selected sgRNA (TTGCGACCACGGCCAGGATC AGG), potential off-target sites were selected.

- Click ‘Show off-target details’.
- Click ‘Export to Excel’ to download the full list of potential off-target sites.
- The most highly predicted top 14 off-target sites were selected for off-target analysis based on the off-target score and number of mismatch and two additional genes in voltage-gated sodium channel (*SCN*) gene family (*SCN8A* and *SCN9A*).
  1.5 The off-target site sequence was uploaded onto the Nucleotide Blast website (https://blast.ncbi.nlm.nih.gov/Blast.cgi?PROGRAM=blastn&PAGE_TYPE=BlastSearch&LINK_LOC=blasthome) to locate the genomic chromosomal locus.
- ‘Database’: Genomic + transcript database
- Select ‘Human genomic plus transcript (Human G+T)’
- Click ‘BLAST’
- Choose the 100% matched fragment with blue colour in ‘Graphic Summary’, click to review the chromosome location information, if matched with the off-target information in Step 1.4, click ‘Alignment’ to see the details.
- Click ‘Graphics’ to see the sequence of this off-target site, then click ‘Zoom-Out’ to include approximate 1kb in length flanking this short off-target site sequence.
- Click ‘Download’ – ‘Download FASTA’ – ‘FASTA (Visible Range)’
  1.6 The downloaded DNA sequence was uploaded onto Primer-Blast website to design the PCR primers for off-target PCR and sequencing (https://www.ncbi.nlm.nih.gov/tools/primer-blast/).
- ‘PCR Template’: The sequence was downloaded as per Step 1.5.
- ‘Database’: Genomes for selected organisms (primary reference assembly only)
- ‘Organism’: Homo sapiens
- Click ‘Get Primers’
- Select the primers with more than 350bp product size, and at least 100bp of left/right arm.

2. **sgRNA synthesis •** **TIMING 6 hours**
  2.1 sgRNA was synthesized using GeneArt™ Precision gRNA Synthesis Kit. According to the selected sgRNA [5’-(+)TTGCGACCA  GGCCAGGATC(-)-3’ AGG], two forward and reverse overlapping oligonucleotides that contain the target DNA sequence and the Tracr Fragment +T7 Primer Mix were designed and ordered as follows.

Forward: 5’-**TAATACGACTCACTATAG**TTGCGACCA  GGCCAGGATC-3’ (38-nt)

Reverse: 5’-**TTCTAGCTCTAAAAC**GATCCTGGCC  TGGTCGCAA-3’ (35-nt)

***Note:*** *If the gRNA contains a 5’G, it can be removed as the Tracr Fragment contains a 3’G already.*

2.2 The ordered forward and reverse oligonucleotides were re-suspended in TE Buffer (10mM Tris-HCl, 0.1mM EDTA; pH 8.0) respectively to get the stock solution with a concentration of 100μM. Ten microliter of each 100μM forward and reverse stock oligonucleotide solution were added into 80μl nuclease-free H_2_O to form a 10μM stock solution of target oligonucleotide mix, which was then diluted to 0.3μM for next step.
2.3 Set up the **sgRNA DNA template synthesis reaction** (25μl) as following:

- Phusion™ High-Fidelity PCR Master Mix (2×) (12.5μl)
- Commercial Tracer Fragment +T7 Primer Mix (1μl)
- 0.3μM Forward and Reverse oligonucleotide mix (1μl)
- Nuclease-free water (10.5μl)

Perform assembly PCR using the cycling below: Initial denaturation 98^o^C 10s (1×); Denaturation 98^o^C 5s, Annealing 55^o^C 15s (32×); Final extension 72^o^C 1min (1×); 4^o^C (Hold).

2.4 Electrophoresis gel of PCR product.

A) Prepare 50×TAE (Tris-Acetate-EDTA) Buffer

- Tris Base (242g)
- Glacial acetic acid (57.1ml)
- 0.5M EDTA solution (pH 8.0) (100ml)
- Adjust volume to 1 L with dH_2_O.

***Optional:*** *One can also use the commercial 50× TAE Buffer and dilute to 1× with dH*_*2*_*O.*

B) Make 1.5% (w/v in 1× TAE Buffer) Agarose gel with SYBR Safe DNA Gel Stain (0.01% v/v).
C) Take 5μl of the PCR product and mix with 1μl of Gel Loading Dye (6×) and loaded for electrophoresis with a 100bp DNA Ladder.

***Note:*** *The expected band should be around 120bp size.*

2.5 Set up ***in vitro*** **Transcription (IVT)** (20μl) in the following order:

- NTP mix (100mM each of ATP, GTP, CTP, UTP) (8μl)
- gRNA DNA template (directly from step 1) (6μl)
- 5× TranscriptAid™ Reaction Buffer (4μl)
- TranscriptAid™ Enzyme Mix (2μl)

Place at 37^o^C and incubate for 3 hours.

***Note:*** *The reaction volume can be doubled if more sgRNA is required. The incubation time can be extended by up to 4 hours if higher sgRNA yields are required.*

2.6 One microliter of DNase I (1U/μl) was added into the reaction mix for digesting DNA template and oligos at 37^o^C for 15 min.

***Note:*** *A white precipitate will form after IVT step, and this contains pyrophosphate and smaller amounts of RNA. However, this will not affect the following purification.*

2.7 Then sgRNA was purified using gRNA Clean Up Kit. Briefly, the volume of IVT reaction (20μl) was adjusted to 200μl with nuclease-free water (180μl), and then mixed thoroughly with 100μl of Binding buffer. One volume (300μl) of 96% ethanol was added to the mix and then transferred into GeneJET^TM^ RNA Purification Micro Column to purify the sgRNA. The purified sgRNA was eluted in 20μl of nuclease-free water.

***Note:*** *The purification steps should be performed at room temperature. An additional empty centrifuge step is required, as the residual ethanol (in wash buffer) may inhibit the RNA purification.*

2.8 The quality of sgRNA was determined by mixing with RNA Loading Dye (2×) Solution and heating at 70^o^C for 10 min, and then running on 2% electrophoresis with a Low Range ssRNA Ladder thereafter.

***Note:*** *The expected band should be around 100-nt.*

2.9 The concentration of sgRNA was measured by using Nanodrop 2000 Spectrophotometer, and then stored at -80^o^C until required.

3. ***In vitro*** **digestion with RNP •** **TIMING 6 hours**

We recommend performing *in vitro* digestion of the sgRNA before electroporation.

3.1 The *in vitro* effect of CRISPR/Cas9 system was validated by incubating RNP complex (consist of sgRNA and Cas9 protein) and the DNA fragment containing the cut site. Firstly, genomic DNA was isolated from control hiPSCs using DNeasy Blood & Tissue Kit. Briefly, the cell pellet was pre-treated with Proteinase K and RNase A, lysed in Buffer AL, then mixed with ethanol and transferred into DNeasy Mini Spin Column. The isolated DNA was eluted in Buffer AE (10mM Tris-HCl, 0.5mM EDTA; pH 9.0).
3.2 A PCR reaction (20μl) was set up to amplify the DNA fragment as following:

- AllTaq Master Mix (2×) (5μl)
- Primer Forward (10μM) (0.5μl)
- Primer Reverse (10μM) (0.5μl)
- Genomic DNA template (50ng/μl) (2μl)
- Nuclease-free H_2_O (12μl)

Perform PCR assembly using the cycling below: Initial PCR activation 95^o^C 2min (1×); Denaturation 95^o^C 5s, Annealing 60^o^C 15s, Extension 72^o^C 10s (40×); 4^o^C (Hold).

3.3 The PCR product was purified using The QIAquick PCR Purification Kit. Briefly, the PCR reaction was mixed with 5 volumes Buffer PB (containing pH indicator I and 3M sodium acetate) and then transferred into a QIAquick Column to purify the PCR product. The purified DNA fragment was eluted in 50μl of Buffer EB (10 mM Tris-HCl; pH 8.5).

***Note:*** *The 3M Sodium acetate was added to adjust the pH to allow the DNA bind to the membrane of the column.*

3.4 To form the RNP complex *in vitro*, the reaction was assembled as following:

- Nuclease-free water (20μl)
- NEBuffer r3.1 (10×) (3μl)
- 300nM sgRNA (3μl)
- 1μM Cas9 protein (1μl)

Incubate at 25^o^C for 10min.

3.5 Then 3μl of 30nM purified Substrate DNA from Step 3.3 was added, mixed thoroughly, and incubated at 37^o^C for 1 hour.

***Note:*** *The converting ng/μl to nM of double-stranded DNA was based on the formula below:*

*Concentration in nM = (Concentration in ng/μl) / (660g/mol × PCR product size in bp) × 10^* ^*6*^

3.6 One microliter of Proteinase K was added and mixed thoroughly and then incubated at room temperature for 10min.
3.7 The fragment analysis was performed on 1.5% Agarose gel.

***Note:*** *Two small fragments can be observed after Cas9 treatment, and the sum of the size should be the same as the PCR product.*

4. **Preparation of hiPSCs (feeder-free, Xeno-free condition) •** **TIMING 7 days**

Prepare hiPSCs cultured in a 6-well plate with Geltrex coating and Essential 8 Flex Medium (E8) as below. The cell lines used were https://hpscreg.eu/user/cellline/edit/NUIGi038-B [35] and https://hpscreg.eu/user/cellline/edit/NUIGi018-A.

***Note:*** *It is recommended to perform at least 2 passages after cell thawing before progressing to electroporation.*

4.1 **Thawing of hiPSCs**

A) We added 500μl of Geltrex into 50ml Knock-Out DMEM and mixed them to get the 1× Geltrex solution (1:100 diluted).

***Note:*** *The diluted Geltrex solution can be kept in 4*^*o*^*C up to 4 weeks.*

B) For coating 2 wells of a 6-well plate, we added 1ml of Geltrex solution into each well.
C) The plate was placed at 37^o^C, 5% CO_2_ incubator for 1-2 hours.
D) Transfer a vial of cryopreserved hiPSCs from liquid nitrogen into a 37^o^C water bath and quickly thaw the cells until only a sliver of ice remains.

***Note:*** *It is important to thaw cell rapidly to minimize any damage to the cell membrane. Don’t loosen the vial during the thawing in the water bath as this may increase the risk of contamination.*

E) Spray the vial with 70% ethanol and then transfer to a biological safety cabinet.
F) Use a 1ml filter tip to transfer the contents from the vial to a 15ml tube with 9ml of E8 media supplementary with 10μM of Y-27632 (E8Y).

***Note:*** *The addition of ROCK inhibitor Y-27632 is important to improve the survival rate of PSC recovery* [36].

G) Centrifuge at 200×g for 4min and aspirate the supernatant.
H) Re-suspend the cell pellet in 2ml of E8Y media and seed onto the Geltrex-coated cell culture plate.
I) Move the plate in several quick, short, back-and-forth and side-to-side motions to distribute the cell aggregates. Return the plate to the incubator.
J) Media should be switched to E8 media after 24 hours, and refresh every day, until at 70~80% confluency.

***Note:*** *Ideally, the cells can reach 70~80% confluency in 3~4 days and can be passaged.*

4.2 **Passaging of hiPSCs**

A) Aspirate media from the cultured hiPSCs.
B) Rinse the cells by adding 1ml of DPBS (without Ca^2+^ or Mg^2+^), gently rock the plate and aspirate the DPBS.
C) Add 400μl of Gentle Cell Dissociation Reagent (GCDR) and incubate at room temperature for 6~8min until the gaps appear between the cells located on the edge of the colonies.

***Note:*** *GCRD is an enzyme-free reagent suitable for the dissociation of hiPSCs into cell aggregates for routine passaging. The dissociation should be monitored under the microscope until the optimal time is determined based on appearance.*

D) Aspirate the GCDR and then add 2ml of E8 media to detach the cell aggregates.

***Note:*** *Avoid pipetting cells too much as it may decrease the cell survival after passaging.*

E) Transfer the cell aggregates into Geltrex-coated 6-well plate with appropriate ratio.
F) Gently move the plate to distribute the cell aggregates. Place the plate in a 37^o^C, 5%CO_2_ incubator.
G) The next day, change media with 2ml of E8 media.
H) Refresh media every other day.
I) IPSCs will be at 70~80% confluency on day 3~4, and ready for performing another passage.

5. **Electroporation using Neon Transfection System •** **TIMING 4 hours**
  5.1 IPSCs with approximately 70% confluency were treated with Y-27632 (ROCK inhibitor) for 2 hours at a final concentration of 10μM.

***Note:*** *The ROCK inhibitor may result in a spindle-shaped cell morphology, however, this change is temporary and will reverse after ROCK inhibitor is removed.*

5.2 IPSCs were dissociated into single cell using 0.5× TrypLE Select (1:1 diluted in DPBS without Ca^2+^ or Mg^2+^) and then re-suspended in E8 media with 1% (v/v) RevitaCell Supplement (E8R) [37].

***Note:*** *It is very important to dissociate into single cells as the cell aggregate may affect the electroporation efficiency.*

***Optional:*** *CloneR (STEMCELL Technologies, #05888) also showed very high efficiency in colony survival in our study.*

5.3 The synthesized sgRNA and Cas9 protein were diluted in TE Buffer to the final concentration of 10μM (800ng and 3.2μg, respectively).

***Note:*** *The size of Cas9 Protein is 160kDa, so 10μM of Cas9 is 10(μM) × 160(kDa) = 1,600μg/ml = 1.6μg/μl.*

5.4 Set up the materials below to form the ribonucleoprotein (RNP) complex:

- sgRNA (10μM, 800ng) (2μl)
- TrueCut Cas9 Protein (10μM, 3.2μg) (2μl)
- Buffer R (6μl)

Gently mixed and incubated at room temperature for 10~15min [38, 39].

***Note:*** *The volume of RNP complex should not exceed 10% of the electroporation reaction.*

5.5 1×10^^6^ hiPSCs were spun down and re-suspended in 100μl of Buffer R.
5.6 Then the electroporation reaction was set up as following:

- RNP complex (10μl)
- Cell suspension (0.2×10^^6^ hiPSCs) (20μl)
- ssODN template (10μM) (10μl)
- Buffer R (70μl)
  5.7 Transfer 100μl of electroporation mix into a Neon 100μl tip very carefully using the Neon Pipette.

***Note:*** *Take care when transferring the electroporation mix and avoid air bubbles in the pipettes as it may cause arcing during electroporation and thus lead to failed transfection.*

5.8 Select Programme ‘1300V, 30ms, 1pulse’ [40], and press ‘Start’.

***Note:*** *It is very important to ensure the metal head of the Neon Pipette is tightly connected to the ball plunger inside of the Neon Pipette Station and to the Neon Tube.*

6. **Subcloning and single cell-derived clone expansion •** **TIMING 12 days**
  6.1 Immediately after electroporation, cells were re-suspended in E8R media.
  6.2 Single hiPSC’s were manually picked and seeded directly into Geltrex-coated 96-well plate.
  6.3 Fifteen to eighteen hours later, microscopic examination was carried out and the wells with only one hiPSC inside were labelled to expansion.
  6.4 The culture media was changed to E8 media two days after electroporation, and refreshed every 2 days.
  6.5 One week later, the single cell-derived colonies can be observed in 96-well plate and then passaged onto a 12-well plate using GCDR for expansion.
  6.6 Four to five days later, the hiPSC colonies were dissociated with GCDR and then ~20% hiPSCs were passaged onto a new 12-well plate for sub-culturing. The remaining ~80% cells were collected for genomic DNA extraction [41].

7. **On-target PCR screening •** **TIMING 6 hours**
  7.1 Genomic DNA was isolated from the single hiPSC-derived colonies (from Step 6.6).
8. Collect cell pellet.

***Optional:*** *Cell pellet could be frozen in -80*^*o*^*C until DNA isolation.*

B) Re-suspend cell pellet in 200μl of DPBS (without Ca^2+^ or Mg^2+^).
C) Add 20μl Proteinase K and 4μl RNase A (100mg/ml), incubate at room temperature for 2min to remove protein and RNA.
D) Add 200μl Buffer AL, vortex and incubate at 56^o^C for 10 min to lyse the cells, digest protein and RNA.
E) Add 1μl pH indicator, and 10μl of 3M NaOAc until suspension becomes yellow.
F) Add 310μl isopropanol to precipitate DNA, vortex and incubate at room temperature for 5min.
G) Vortex for 30 sec, and then centrifuge at 12,000×g in 4^o^C for 30min. Discard the supernatant.
H) Gently add 500μl of 70% ethanol to wash the DNA pellet, spin at 12,000×g in 4^o^C for 5min. Remove ethanol carefully.
I) Quick spin at 12,000×g, and remove ethanol as much as possible using a 10μl tip.
J) Air dry the DNA pellet at room temperature overnight.
K) Resuspend DNA pellet with 20~50 μl Buffer AE and ready for PCR amplification.

***Note: This method showed similar effectiveness and lower cost compared with genomic DNA isolation using RNeasy Blood & Tissue Kit (Qiagen).***

7.2 The PCR was assembled and purified based on the protocols in Step 3.2 and 3.3.
7.3 Purified PCR products (5ng/μl, in 15μl) were submitted to Eurofins Genomics for Sanger sequencing.

8. **Off-target Analysis •** **TIMING 6 hours**
  8.1 The 20-nt sgRNA is designed to direct the Cas9 protein to a particular target location within a specified gene, and the Cas9 is designed to cleave DNA around 3~4bp upstream of the PAM sequence. However, the interaction between the sgRNA and genomic DNA can tolerate some potential alignment mismatch, leading to potential off-target nuclease activity elsewhere in the genome [42, 43].
  8.2 The genomic DNA of the screened colonies with successful editing in Step 8 was assembly using the off-target site primers designed in Step 1.6. (Table 1)

**Table 1:**
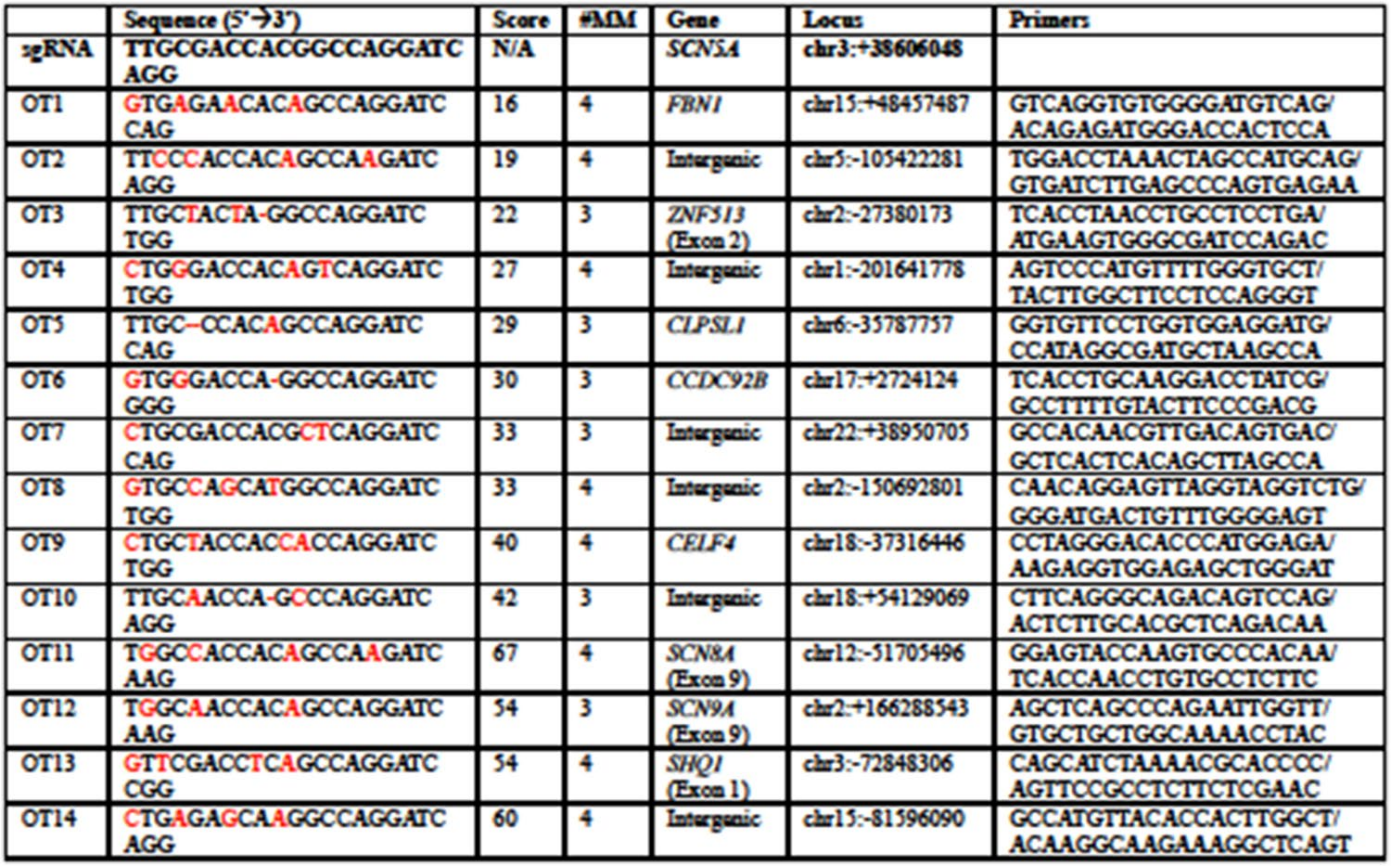
The information of potential off-target sites and the primer design
  8.3 The purified PCR products were submitted to Eurofins Genomics for Sanger sequencing.

9. **Characterization of Genome edited hiPSC clones •** **TIMING 1 month**
  9.1 The genome edited hiPSC clones were expanded culture in E8 media based on the protocol in Step 4.
  9.2 **Cryopreservation of hiPSCs**

When cells reached 70~80% confluency, we cryopreserved a proportion of cells for biobanking.

A) Aspirate the culture media and add 1ml of E8Y media.

***Note:*** *The pre-treatment of ROCK inhibitor can help increase the survival rate of hiPSCs after cryopreservation / thawing.*

B) Incubate at 37^o^C, 5%CO_2_ incubator for 1 hour.
C) Detach the cells using Cell Scraper, and then transfer all the cells into a 15ml centrifuge tube.
D) Centrifuge at 200×g for 4min and aspirate the supernatant.
E) Re-suspend the cell pellet in 1ml of cryopreservation media (90% Knock-out Serum Replacement (KOSR) and 10% DMSO).

***Note:*** *KOSR is an animal-free and xeno-free media.*

F) Put the cryovial in the Thermo Scientific™ Mr. Frosty™ freezing container and leave in the -80^o^C freezer overnight.

***Note:*** *After re-suspending cells in cryopreservation media, transfer the cryovial into -80*^*o*^*C as quick as possible. The cell viability may decrease with the long-term maintenance in cryopreservation media (containing DMSO) at room temperature.*

G) The next day, transfer the cryovial in the liquid nitrogen tank.

***Note:*** *Long-term storage in the -80*^*o*^*C freezer may also decrease the viability of the hiPSCs post thawing.*

9.3 The alkaline phosphatase activity was analysed using Alkaline Phosphatase Staining Kit II. Briefly, the day 3 hiPSCs were fixed and washed with DPBS (containing 0.05% Tween-20), then incubated with fresh prepared AP substrate solution in the dark at room temperature for 10~15min until image capture.
9.4 Genomic DNA was extracted for molecular karyotyping array to detect copy number variant (CNV) and short tandem repeat (STR) analysis.

***Note:*** *The genomic DNA extraction should include a pre-treatment with RNase A and Proteinase K to remove RNA and protein.*

***Optional:*** *The STR analysis can be performed as Cell Line Authentication (CLA) in Eurofins Genomics, also electrophoresis gel of amplified PCR product on 16 loci sites using our designed primers* [35].

9.5 The expression of pluripotency genes were assessed by quantitative real-time polymerase chain reaction (qRT-PCR), and the pluripotency proteins were evaluated by immunofluorescence staining.

A) RNA was extracted using RNeasy Mini Kit. Briefly, hiPSCs were collected and lysis in Buffer RLT, and then mixed with 1 volume of 70% ethanol. The homogenized lysate was then transferred into RNeasy spin column. The isolated RNA was eluted in DNase/RNase-free H_2_O.
B) One microgram of RNA used to synthase cDNA using QuantiNova Reverse Transcription Kit. Firstly, the potential genomic DNA in the RNA samples were removed by incubated with genomic DNA Removal Mix, and then the reverse transcription (RT) reaction was performed with the RT enzyme and mix provided in the kit.
C) Quantitative real-time PCR (qRT-PCR) was performed to assess the mRNA expression levels of pluripotency genes with SYBR Green Master Mix, house-keeping gene GAPDH was used as the internal control.
  9.6 The spontaneously differentiation capacity was assessed by *in vitro* embryonic body (EB) formation with cells in three germ layers endoderm, mesoderm, and ectoderm.
D) The hiPSCs were dissociated and spun down in AggreWell 400 Plate and culture 24 hours to form EBs.
E) The EBs were transferred to non-coated 6-well plate and cultured in suspension for 7 days in EB media:

- DMEM/F-12, GlutaMAX Supplement 384ml.
- KOSR 100ml, Final: 20%
- L-Glutamine 5ml, Final: 1%
- MEM Non-Essential Amino Acid Solution (NEAA) 5ml, Final: 1%
- Penicillin-Streptomycin 5ml, Final: 1%
- β-mercaptoethanol 1ml, Final: 0.2%

III) Individual EBs were picked up and seeded in Geltrex-coated chamber slides and cultured for spontaneous differentiation for 4 weeks.
IV) On day 28, the cells were fixed and performed immunofluorescence staining with three germ layer markers: SRY-Box Transcription Factor 17 (SOX17) for endoderm, alpha-actin smooth muscle (α-SMA) for mesoderm, and β3-tubulin (TUJ1) for ectoderm.
V) Images were acquired by FluoView 1000 Confocal Microscope.

10. **Cardiomyocyte Differentiation from hiPSCs •** **TIMING 14 days**
  10.1 On day -1, hiPSCs in 6-well plate were treated in E8Y for 2 hours and then dissociated into single cells using GCDR.

***Note:*** *Using high quality hiPSCs (with minimal or no differentiated colonies) is very important.*

10.2 3.5×10^^5^ (350k) hiPSCs were seeded on Geltrex-coated 12-well plate and incubated with E8R media at 37^o^C for 24 hours to reach 90% confluency.

***Note:*** *The start seeding cell density is very critical for an efficient cardiomyocyte differentiation from hiPSCs. We recommend start cardiac differentiation with a cell density of 80~90% confluency within 24~48 hours after plating.*

10.3 PSC Cardiomyocyte Differentiation Medium A was applied on differentiation day 0 and Medium B on day 2. From day 5, Cardiomyocyte Maintenance Medium (CMM) was applied and refreshed every other day.

***Note:*** *Keep all the cardiac differentiation media in 4*^*o*^*C. Shedding of dead cells on Day 2 and day 5 is normal.*

10.4 On differentiation day 14, spontaneously beating clusters of cardiomyocytes were manually dissected and then incubated at 37^o^C with STEMdiff™ Cardiomyocyte Dissociation Medium for 10min to dissociate the cardiomyocyte aggregate into single cells.

***Note:*** *The dissociation can be stopped by adding the Cardiomyocyte Support Medium.*

10.5 Centrifuge at 300×g for 5min.
10.6 The cell pellet was re-suspended in STEMdiff™ Cardiomyocyte Support Medium (CSM) containing 1% RevitaCell Supplement for downstream work.

11. **Cardiomyocyte characterization •** **TIMING 14 days**
  11.1 A density of 50k/200μl cardiomyocytes were seeded on Matrigel (1:100 (v/v) diluted in KnockOut DMEM)-coated 24-well cell culture plates for RNA extraction and 10k/150μl cardiomyocytes 8-well chamber slides for immunofluorescence staining.
  11.2 The culture media was switched to CMM after 24 hours and refreshed every other day thereafter.
  11.3 On differentiation Day 28, the hiPSC-CMs were washed with cold DPBS (without Ca^2+^ or Mg^2+^), spun down, and the RNA was isolated using RNeasy Mini Kit. Then the qRT-PCR was performed to assess the expression of cardiac genes.
  11.4 The cardiomyocytes in chamber slides were fixed and performed immunofluorescence staining to assess the cardiac markers.

12. **LQT phenotype using Multi-electrode Array •** **TIMING 14 days**
  12.1 The Axion BioSystems CytoView MEA 48 Plate was coated for 2 hours, with 50μg/ml of Fibronectin (by diluting in DPBS with MgCl_2_ and CaCl_2_).

***Note:*** *Sterile H*_*2*_*O or DPBS (without Ca*^*2+*^ *and Mg*^*2+*^*) can be added to the on-plate reservoirs to increase humidity and prevent fibronectin drying out during the incubation.*

12.2 On Day 14, dissociated cardiomyocytes were seeded on Fibronectin-coated MEA plate (at a density of 1×10^^4^/10μl) and cultured with CSM containing 1% RevitaCell Supplement for 24 hours.

***Note:*** *Timing is critical in this step. Adding a drop of cardiomyocytes on MEA plates for ~2 hours to ensure the cardiomyocyte attachment.*

12.3 The culture media was switched to CMM and refreshed every other day.

***Note:*** *Avoid touching the electrodes when refreshing the media.*

12.4 The field potential was recorded daily from Differentiation Day 17 (3 days after replating on MEA plate) using AxIS Navigator software (Version 2.5.1.10) with the recommended settings below.

- ‘Streams – Configuration – Cardiac Real-time – Spontaneous’
- Digital Filter: 0.1Hz on high pass and 2,000Hz on low pass.
- The threshold for detecting cardiac beat: 200μV.
- Minimum and maximum beat period at 250ms and 10s (beat rate between 6 and 240 beat per minute (BPM)).
- FPD Method: Polynomial Regression (calculate the field potential duration with maximal post search duration of 1s, and the pre- and post-spike detection holdoff at 50ms and 70ms respectively).
  12.5 Temperature was monitored during measurement and kept at 37^o^C for a stable ion channel function and to prevent temperature-dependent dysregulation of ion channels [44].

***Note:*** *On the day of media refreshment, cardiomyocytes were equilibrated for at least 1 hour at 37*^*o*^*C and 5% CO*_*2*_ *before recording field potential.*

12.6 After recording, the primary recorded AxIS Raw data was analysed in CiPA Analysis Tool (Version 3.1.8) for scientific analysis. Only one electrode, which showed a significant positive or negative T-wave and reflected the signal of most electrodes in each well, was selected as the Golden Channel from each well to detect the field potential duration (FPD) [45]. The corrected FPD (FPDc) was calculated using Fredericia’s correction formula [46].

## Anticipated Result

Immediately after electroporation, we picked a total of 192 single iPSCs and seeded into two 96-well plates. On the second day, we identified 91 wells with single cell survival (cell survival rate as 47.40%). One week later, colonies were observed in 69 wells (clone formation efficiency as 75.82%). After Sanger sequencing from the genomic DNA of these 69 colonies, 6 clones were confirmed to have successfully incorporated the desired heterozygous variant of interest introduced (HRD efficiency as 8.70%) (Fig. S1). Following this, off-target analysis of the most likely (top 14) off-target DNA sites was performed on these 6 colonies and that result showed no off-target effect detected (Fig. S2). We acknowledge the HDR efficiency could be further improved in future work, with such strategies as optimizing the Cas9/gRNA ratio, introducing synonymous variants and a silent restriction digestion site in the ssODN design.

Furthermore, the general characterization confirmed that all these 6 edited hiPSC clones showed normal karyotype, positive pluripotency markers (OCT4, SOX2, NANOG, SSEA4, TRA-1–60 and TRA-1–81) and the spontaneous differentiation capacity to three germ layers. We subsequently differentiated the hiPSC clones into a cardiac lineage and measured the electrophysiological characteristics of the resultant cardiomyocytes using a multi-electrode array system.

In summary, we provide an RNP-based CRISPR/Cas9 strategy to introduce a heterozygous variant in wild-type hiPSCs for subsequent differentiation into a cardiac lineage for further pathogenic phenotyping. This approach can also facilitate the generation of isogenic control lines from the patient-derived hiPSCs, which represents a gold standard of control when investigating a single nucleotide variable in a disease genotype–phenotype comparison. A combination of CRISPR/Cas9 techniques and stem cell modelling can be used for *in vitro* disease modelling, phenotype comparison, pharmacological exploration, drug development and future personalized therapy.

### Supplementary Information

Below is the link to the electronic supplementary material.

Supplementary file1 (PDF 6526 kb)

Supplementary file2 (PDF 6127 kb)

Supplementary file3 (MP4 36409 kb)

Supplementary file4 (MP4 27785 kb)

## Data Availability

None.
