# Supplementary material for: Using Ribonucleoprotein-based CRISPR/Cas9 to Edit Single Nucleotide on Human Induced Pluripotent Stem Cells to Model Type 3 Long QT Syndrome (SCN5A±)
Source: Stem Cell Rev Rep. 2023 Aug 31;19(8):2774–89. doi: 10.1007/s12015-023-10602-5 (PMC10661835; doi:10.1007/s12015-023-10602-5)
Supplement: Supplementary file 1 — Supplementary file1 (PDF 6526 kb) [file 12015_2023_10602_MOESM1_ESM.pdf]

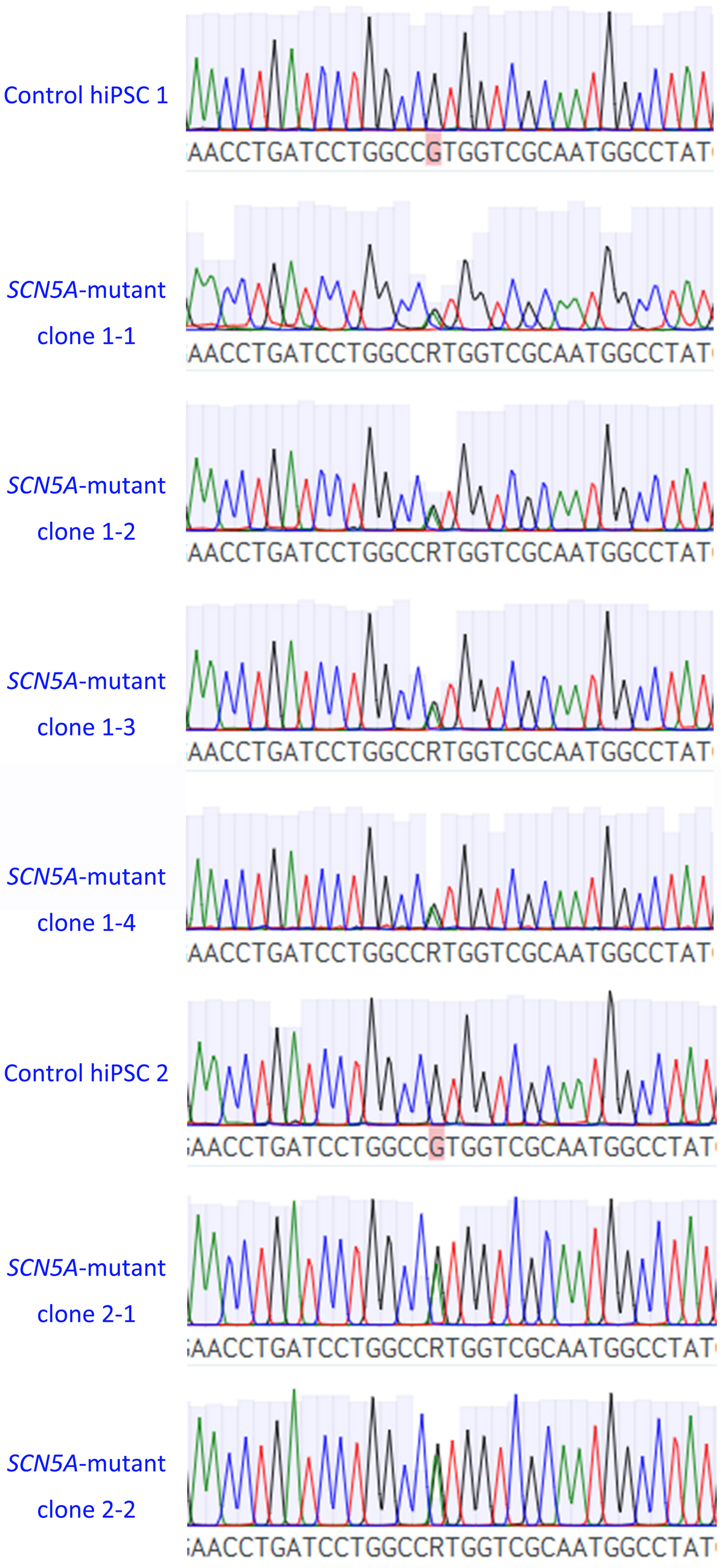

Control hiPSC 1
AACCTGATCCTGGCCGTGGTCGCAATGGCCTAT
SCN5A-mutant clone 1-1
AACCTGATCCTGGCCRTGGTCGCAATGGCCTAT
SCN5A-mutant clone 1-2
AACCTGATCCTGGCCRTGGTCGCAATGGCCTAT
SCN5A-mutant clone 1-3
AACCTGATCCTGGCCRTGGTCGCAATGGCCTAT
SCN5A-mutant clone 1-4
AACCTGATCCTGGCCRTGGTCGCAATGGCCTAT
Control hiPSC 2
AACCTGATCCTGGCCGTGGTCGCAATGGCCTAT
SCN5A-mutant clone 2-1
AACCTGATCCTGGCCRTGGTCGCAATGGCCTAT
SCN5A-mutant clone 2-2
AACCTGATCCTGGCCRTGGTCGCAATGGCCTAT
