# Supplementary figures and images for: Using Ribonucleoprotein-based CRISPR/Cas9 to Edit Single Nucleotide on Human Induced Pluripotent Stem Cells to Model Type 3 Long QT Syndrome (SCN5A±)
Source: Stem Cell Rev Rep. 2023 Aug 31;19(8):2774–89. doi: 10.1007/s12015-023-10602-5 (PMC10661835; doi:10.1007/s12015-023-10602-5)

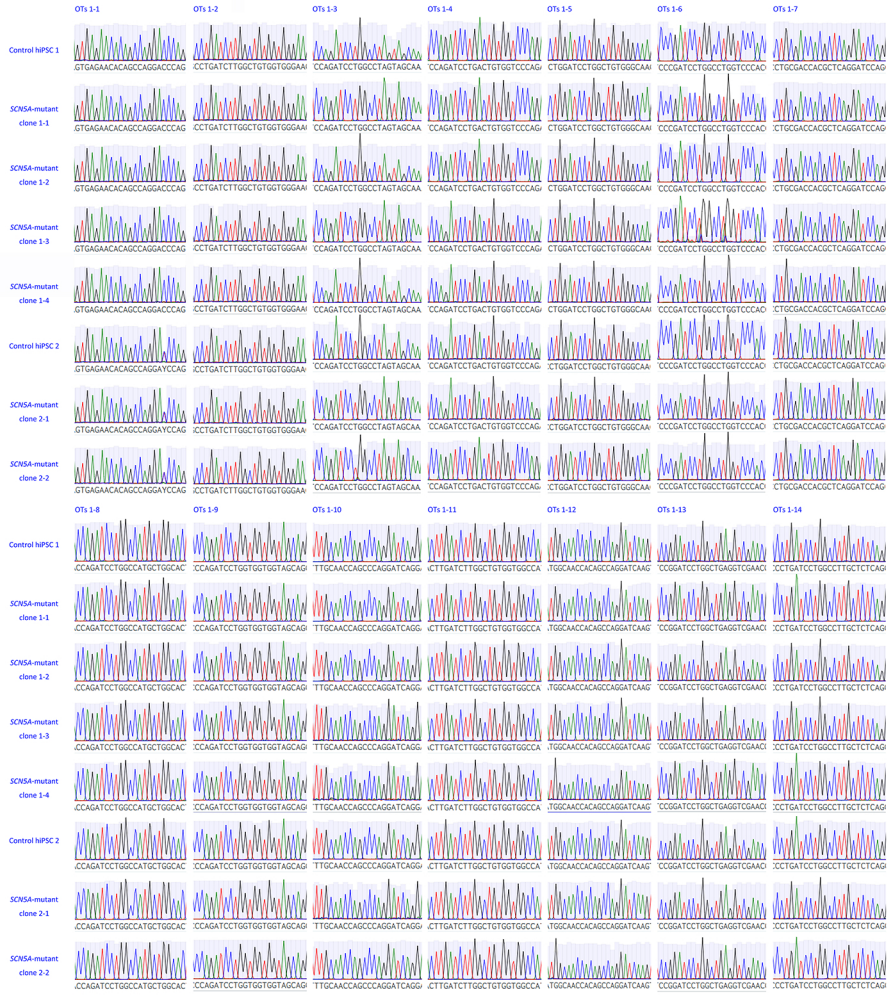

Supplement: Supplementary file 2 — Supplementary file2 (PDF 6127 kb) [file 12015_2023_10602_MOESM2_ESM.pdf]
